# Caries Detection Methods Based on Changes in Optical Properties between Healthy and Carious Tissue

**DOI:** 10.1155/2010/270729

**Published:** 2010-03-28

**Authors:** Lena Karlsson

**Affiliations:** Division of Cariology, Department of Dental Medicine, Karolinska Institutet, Box 4064, 141 04 Huddinge, Sweden

## Abstract

A conservative, noninvasive or minimally invasive approach to clinical management of dental caries requires diagnostic techniques capable of detecting and quantifying lesions at an early stage, when progression can be arrested or reversed. Objective evidence of initiation of the disease can be detected in the form of distinct changes in the optical properties of the affected tooth structure. Caries detection methods based on changes in a specific optical property are collectively referred to as optically based methods. This paper presents a simple overview of the feasibility of three such technologies for quantitative or semiquantitative assessment of caries lesions. Two of the techniques are well-established: quantitative light-induced fluorescence, which is used primarily in caries research, and laser-induced fluorescence, a commercially available method used in clinical dental practice. The third technique, based on near-infrared transillumination of dental enamel is in the developmental stages.

## 1. Introduction

Dental caries is one of the most prevalent chronic diseases of humans worldwide. When different stages of the disease are taken into account, from the initial to the clinically manifest lesion, very few individuals are truly unaffected. In most industrialised countries 60%–90% of school-aged children are affected. The prevalence among adults is even higher and in most countries the disease affects nearly 100% of the population [1]. 

 During the last thirty years, however, major changes have occurred in the pattern of the disease. Progression of enamel caries is now slower [2], allowing time for preventive intervention before irreversible destruction of tooth substance occurs. During the early stages of the disease the process is reversible and can be arrested: noninvasive intervention can convert a lesion from an active to an inactive state [3, 4]. Appropriate diagnostic techniques are necessary to support such decisions about management of the individual lesion. The clinician needs to be able to monitor the outcome of noninvasive measures and in cases where there is evidence of lesion progression, make a timely decision to intervene, using minimally invasive techniques and restoring damaged tooth structure without weakening the tooth. Applying strategies to control, arrest, or reverse the disease process can reduce the economic burden, pain, and suffering of placing and replacing restorations [5].

This modern, conservative approach to clinical management of dental caries, which has been evolving during the past twenty years, has necessitated a critical appraisal of methods used today for clinical detection of carious lesions.

Complementing traditional diagnostic methods with advanced, more sensitive methods will improve caries diagnostic routines and hence the dental care and treatment of patients. The application of such complementary methods should offer objective information about the presence and severity of a lesion, to complement the clinician's subjective interpretation, providing evidence-based clinical caries diagnosis. In this context, there is also a place for more sensitive caries detection methods in clinical caries research. Clinical trials in which lesions are monitored in thousands of subjects over several years are no longer commercially viable. A quantitative method capable of measuring small changes would allow trials of much shorter duration and fewer subjects [6, 7]. 

Conventional examination for caries detection is based primarily on subjective interpretation of visual examination and tactile sensation, aided by radiographs. The clinician makes a dichotomous decision (absence or presence of a lesion) based on subjective interpretation of colour, surface texture, and location, using rather crude instruments such as a dental explorer and bitewing radiographs [8]. Studies based on these methods often show low sensitivity and high specificity, that is, a large number of lesions may be missed [9–13]. Sensitivity and specificity are widely used measures to describe and quantify the diagnostic ability of a test [14]. In the context of caries research, sensitivity is a measure of the method's ability to correctly identify all surfaces damaged by caries, and specificity the measure of correctly identified all sound surfaces. Sensitivity and specificity are expressed as values between 0 and 1 (100%), values closer to 1 indicating a high quality result. For caries diagnostic methods, values should be at least 0.75 for sensitivity and over 0.85 for specificity [15]. 

Diagnostic techniques are also evaluated in terms of validity and reliability. To determine validity, the outcome as measured by the method is compared with a reference standard, a ‘‘true” situation. Reliability expresses the consistency of a set of measurements performed with the method. High validity is considered to confirm the absence of systematic errors and high reliability the absence of random errors of the method. The generalisability of a diagnostic technique is also described in terms of external and internal validity. The external validity reflects the extent to which the results of a study can be extrapolated to other subjects or settings, whereas internal validity reflects the degree to which conclusions about causes or relationships are likely to be true, in view of the measures used, the research setting, and the overall study design. Good experimental design will filter out the most confounding variables, which could compromise the internal validity of an experiment. 

A wide variation in terms of sensitivity and specificity for conventional caries detection methods are found in the literature [9, 16, 17]. An overall low sensitivity of less than 0.50 is reported, which means that a guess would provide the same result when we correctly want to identify a caries lesion. A recently published comprehensive review [15] stated that the evaluations of diagnostic performance are based on limited numbers of studies of questionable internal and external validity attributable to incomplete descriptions of selection and diagnostic criteria and observer reliability. The quality of published studies is further compromised by the use of small numbers of observers, nonrepresentative teeth, samples with high lesion prevalence, a variety of reference standards of unknown reliability, and variations in statistical analysis of the reported results.

It is apparent that conventional methods for the detection of dental caries do not fulfill the criteria for an ideal caries detection method. These methods rely on subjective interpretation and are insensitive to early caries detection. It is widely recognised that the current methods cannot detect caries lesions until a relatively advanced stage, involving as much as one-third or more of the thickness of enamel [18]. 

The shortcomings of conventional caries detection methods and the need for supplementary methods have long been acknowledged. The series of published proceedings from the three “Indiana Conferences on Early Detection of Dental Caries” contains a wealth of detail of work in this area [19–21]. Over the past twenty years there has been intensive research into more sophisticated methods for early detection of dental caries [5, 7–9, 16, 17, 22–34]. There are a number of optical caries detection methods and some are summarized in Table 1. Several are in their infancy and there is significant work involved in developing these techniques. Therefore, validation studies are essential to determine their clinical utility before implementation in clinical practice.

An initial effect of the caries disease process, increased porosity, results in a distinct change in the optical properties of the affected dental tissue, providing objective evidence of a caries-induced change. Caries detection methods based on changes in a specific optical property are referred to in the literature as optically based methods, optical methods, or dental tissue optics. The methods are based on the measurement of a physical signal, derived from the interaction of light with dental hard tissue. The following section presents a brief description of the principles underlying these methods.

## 2. Physical Principles Underlying Optical Caries Detection

**Figure 1:**
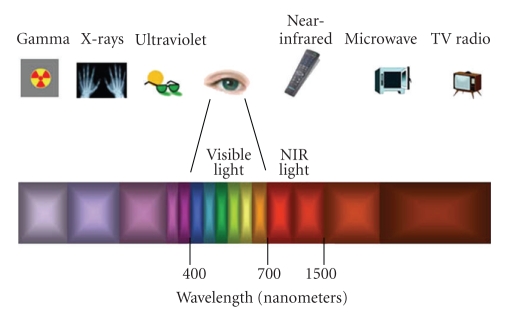
The electromagnetic spectrum.Wavelengths of interest in this paper are the visible light spectrum from 400 nm to 700 nm and the range of near-infrared light from 750 nm to 1500 nm.

Optical caries detection methods are based on observation of the interaction of energy which is applied to the tooth, or the observation of energy which is emitted from the tooth [26]. Such energy is in the form of a wave in the electromagnetic spectrum (Figure 1). The caries detection methods described in this paper use light in the visible and near-infrared range (NIR).

**Figure 2:**
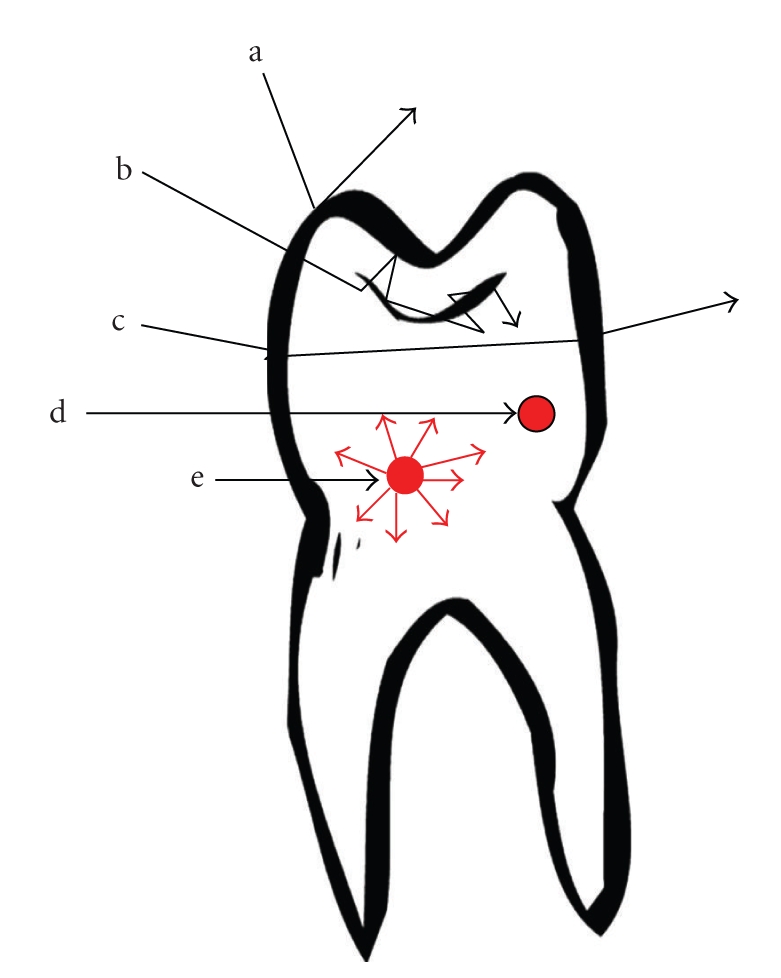
Light interactions with a tooth. How waves can interact with the dental hard tissue; (a) reflection, the wave rebounds; (b) scattering, the incident wave enters the tooth and changes direction. The photons then leave the tooth either as backscattering, where the photons leave through the surface by which they entered, or through another surface (scattering with diffuse transmission); (c) transmission, the wave is illuminated through the tooth and refracts on the surfaces; (d) absorption with heat production; (e) absorption with fluorescence. Most interactions of waves are a combination of these processes.

In its simplest form, caries can be described as a process resulting in structural changes to the dental hard tissue. The diffusion of calcium, phosphate, and carbonate out of the tooth, the demineralisation process, will result in loss of mineral content. The resultant area of demineralised tooth substance is filled mainly by bacteria and water. The porosity of this area is greater than that of the surrounding structure. Increased scattering of incident light due to this structural change appears to the human eye as a so-called white spot. Hence, the caries process leads to distinct optical changes that can be measured and quantified with advanced detection methods based on light that shines on and interacts with the tooth (Figure 2). 

### 2.1. Scattering

Scattering is the process in which the direction of a photon is changed without loss of energy. The incident light is forced to deviate from a straight path when it interacts with small particles or objects in the medium through which the light passes. In physical terms scattering is regarded as a material property. A glass of milk is seen as white because incident light on the milk is scattered in all directions, leaving the milk without absorption [35]. Snow appears white because light incident in the snow is scattered in all directions by the small ice crystals. Light of all visible wavelengths exits snow without suffering absorption. Scattering is highly wavelength sensitive, shorter wavelengths scatter much more than longer ones [26]. Therefore, caries detection methods employing wavelengths in the visible range of the electromagnetic spectra (400 nm to 700 nm) are highly limited by scattering. An early enamel lesion looks whiter than the surrounding healthy enamel because of strong scattering of light within the lesion [23]. Methods measuring lesion severity are based on differences in scattering between sound and carious enamel.

### 2.2. Absorption with Fluorescence

Absorption is the process in which photons are stopped by an object and the wave energy is taken in by the object. The energy lost is mostly converted into heat or into another wave which has less energy and hence longer wavelengths. In physical terms absorption is also regarded as a material property. The previous analogy of the glass of milk appearing white can be extended to a cup of tea [35]; the tea is seen as transparent because it does not scatter light, but it looks brown because much of the light is absorbed by the tea. Likewise, mud and pollution in white snow can be seen as dark spots because certain wavelengths are absorbed by these polluted spots. Absorption of light in tissue is strongly dependent on the wavelength. Water is an example of a strong absorber in the infrared range. After absorption the energy can be released by emission of light at a longer wavelength, through the process of fluorescence. Fluorescence occurs as a result of the interaction of the wavelength illuminating the object and the molecule in this object. The energy is absorbed by the molecule with subsequent electronic transition to the next state, to a higher level state where the electrons remain for a short period of time. From here the electrons may fall back to the ground state and release the gained energy in terms of longer wavelength and colour, which is related to the energy given off and fluorescent light can be emitted. Autofluorescence, the natural fluorescence of dental hard tissue without the addition of other luminescent substances has been known for a long time [36]. Demineralisation will result in loss of autofluorescence [37] which can be quantified using caries detection methods based on the differences in fluorescence between sound and carious enamel.

## 3. Optical Caries Detection Methods 

### 3.1. Quantitative Light-Induced Fluorescence

**Figure 3:**
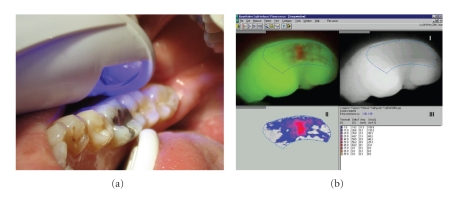
(a) Light of certain wavelengths is led by an optic fibre from the light source to a hand piece with a micro-Charge Couple Device video camera. (b) The image can be captured and saved for later analysis. Computer program: QLF 1.97e Inspector Research System BV, Amsterdam, The Netherlands.

The quantitative light-induced fluorescence (QLF) is based on the principle that the autofluorescence of the tooth alters as the mineral content of the dental hard tissue changes. Increased porosity due to a subsurface enamel lesion scatters the light either as it enters the tooth or as the fluorescence is emitted, resulting in a loss of its natural fluorescence. Bjelkhagen et al., [38], Sundström et al., [39] and subsequently de Josselin de Jong et al. [40] developed a technique based on this optical phenomenon. The underlying theory has been described extensively in several publications [41–43]. The changes in enamel fluorescence can be detected and measured when the tooth is illuminated by violet-blue light (wavelengths 290–450 nm, average 380 nm) from a camera hand piece, following image capturing using a camera fitted with a yellow 520 nm high pass filter (QLF; Inspektor Research Systems, Amsterdam, the Netherlands) (Figure 3(a)). The image is captured, saved, and processed: it is first converted to black-and-white so that thereafter the lesion site can be reconstructed by interpolating the grey level values in the sound enamel around the lesion. The difference between measured and reconstructed values gives three quantities: Δ*F* (average change in fluorescence, %), lesion area (mm^2^), and ΔQ (area ×Δ*F*), which gives a measure of the extent and severity of the lesion. Changes in fluorescence radiance and lesion area can be followed over time, to measure lesion development. Figure 3(b) shows the analytical stages of the method. 

A high positive correlation is reported between QLF and absolute mineral loss, *r* = 0.82–0.92 [44–46]. At a consensus meeting in 2002, The International Consensus Workshop on Caries Clinical Trials (ICW-CCT) [47], it was agreed that QLF may offer one solution in the effort to reduce both the number of subjects and the duration of caries clinical trials. The method seems to have been rapidly adopted as a standard reference measure in clinical tests of the efficacy of preventive measures [42, 48–50]. Application for quantification of dental fluorosis [51], erosive lesions [52, 53], and staining, and bleaching of teeth [54–56] has been investigated. The QLF method can also measure and quantify the red fluorescence (RF) from microorganisms in plaque [57]. The RF observed in plaque can be of use when monitoring oral hygiene, denture plaque assessment, removing infected dentin, and detecting a leaking sealant or caries at the margin of a restoration. Two quantities are obtained, Δ*R* (average change in red fluorescence, %) and area (mm^2^). So far there are a very limited number of studies performed with this feature.

### 3.2. Laser-Induced Fluorescence

**Figure 4:**
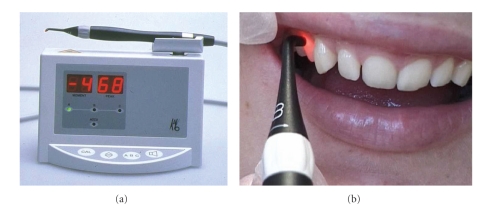
(a) The LF device operates with light from a diode laser transmitted through a descendent optic fibre to a hand held probe with a fibre optic eye. The emitted fluorescence is collected through the tip, passes into ascending fibres, and is finally processed and presented on the display as an integer between 0 and 99. (b) In the presence of carious tooth substance, fluorescence increases.

The DIAGNOdent (KaVo, Biberach, Germany) is a portable commercially available device (Figure 4(a)) for detection and quantification of caries [28, 58]. The method generates a simple numerical index of de- and remineralisation in enamel and dentin that can be recorded in the patient's file and monitored over time. The instrument is easy to handle and can also be purchased at a reasonable price. Red laser light (*λ* = 655 nm) is emitted by the device via an optical fibre and a probe to the caries lesion (Figure 4(b)). When the light interacts with certain organic molecules that have been absorbed into the porous structure the light is reemitted as invisible fluorescence in the NIR region. The NIR fluorescence is believed to originate from protoporphyrin IX and related metabolic products of oral bacteria [28, 59]: these products are chiefly responsible for the absorption of red light. The emitted light is channelled through the hand piece to the detector and digitally displayed on a screen (0–99). A higher number indicates greater fluorescence and by inference a more extensive subsurface lesion. 

Two versions of the laser fluorescence (LF) device are currently available commercially. As well as the DIAGNOdent 2095 for application to smooth and occlusal surfaces, the latest version, the LF-pen (KaVo), has been designed for easier access to approximal surfaces. The original LF device has shown good performance and reproducibility for detection and quantification of occlusal and smooth surface caries lesions in in vitro studies, but the results of in vivo studies have been somewhat contradictory [60–70]. Among LF studies there is a wide variation in specific design features (the number of teeth included, the threshold for LF scores, validation methods, nonvalidated teeth, the outcomes expressed, etc.). A review by Bader and Shugars [24] disclosed that although several evaluations of diagnostic performance have appeared in the literature, the range of the LF device performances is extensive. For detection of dentinal caries, sensitivity values ranged widely (0.19 to 1.0) although most tended to be high. Specificity values exhibited a similar pattern, ranging from 0.52 to 1.0. In comparison with visual assessment methods, the LF exhibited a sensitivity value that was almost always higher and a specificity value that was almost always lower. The body of evidence was based primarily on in vitro studies. Extrapolation to the clinical setting is uncertain. 

The LF pen has performed as well as the original device on occlusal surfaces in vitro [71]. To date, there is only one published study of the clinical performance of the LF pen on occlusal surfaces [72]. A moderately positive correlation (Spearman rho) was demonstrated, and greater variation of measurements was recorded with increasing clinically evaluated lesion depth. At a cut-off value of 25 for the threshold between enamel and dentinal caries, sensitivity was 0.67 and specificity was 0.79. 

The LF method has also been investigated for longitudinal monitoring of the caries process and for assessing the outcome of preventive interventions [50, 63, 73–75]. The potential role of the LF device in detection of root caries lesions has not been extensively investigated and hitherto only three validity studies are available [76–78]. A low to moderate correlation was found when LF readings were correlated with histopathological lesion depth of root caries lesions [76, 77].

For the clinician to have confidence in using a caries detection method to support clinical treatment decisions, it is important that interpretation of readings is based on an understanding of the principles underlying the method and an awareness of potential shortcomings. With reference to LF, the question of what the method really measures has yet to be resolved. More research is needed to clarify the origin of the increased fluorescence caused by the excitation of 655 nm wavelength light. The NIR fluorescence is believed to originate from bacteria or their metabolites. Hence, there is a poor correlation between LF readings and the mineral content, but possibly better correlation with the presence of infected dentin. 

In general, in vivo studies of LF for occlusal caries detection indicate moderate to high sensitivity and lower specificity [24, 60, 66, 68]. Lack of specificity, the increased likelihood of false-positive readings due to stain and plaque, and the absence of a single threshold are factors underlying the reluctance among authors to recommend the LF method unequivocally for caries detection. Therefore, the LF device should be regarded at most as a supplementary aid for detection of caries on coronal surfaces.

### 3.3. Transillumination with Near-Infrared Light

**Figure 5:**
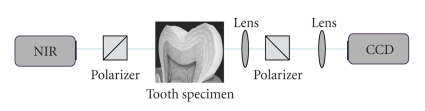
Transillumination (TI) with Near-Infrared (NIR) light. experimental set-up of the TI system. The tooth is illuminated with NIR light. Polarizers are used to experimentally block out the ambient light from saturating the detector, a Charge Couple Device (CCD).

The caries lesion may also be examined by shining white light through the tooth. Wavelengths in the visible range (400–700 nm) are limited by strong light scattering, making it difficult to image through more than 1 mm or 2 mm of tooth structure [79]. Therefore, methods employing wavelengths in the visible range of the electromagnetic spectra (400–700 nm) such as QLF [40] (*λ* > 520 nm), LF [58] (*λ* = 655 nm), and Digital Imaging Fiber-Optic Transillumination (DIFOTI) [80]—which uses high intensity white light—are highly limited by scattering. Methods that use longer wavelengths, such as in the NIR spectra (780 to 1550 nm), can penetrate the tissue more deeply. This deeper penetration is crucial for the transillumination (TI) method. Research has shown that enamel is highly transparent in the NIR range (750 nm to 1500 nm) due to the weak scattering and absorption in dental hard tissue at these wavelengths [81–86]. Therefore, this region of the electromagnetic spectrum is ideally suited to the development of new optical diagnostic tools based on TI. Figure 5 illustrates the typical experimental set-up of a TI system with an NIR light source, an imaging camera such as a charge-coupled device (CCD), and software for computer-controlled acquisition. The image can be captured, saved, and stored in digital format.

This is a promising imaging technique for detecting the presence of caries and measuring its severity. The TI image is presented as a visually recognizable image, which is preferred by the average clinician. The method is nondestructive, nonionising, and reportedly more sensitive to detect early demineralisation than dental *X*-rays [83]. Identification of dental caries by TI is based on the fact that increased mineral loss in an enamel lesion leads to a twofold increase in scattering coefficient at a wavelength of 1.3 *μ*m [82, 83]. Caries thus appear as dark regions, since less light reaches the detector. Most research to date has used this wavelength, where low-cost light sources are available. When light illuminates the tooth the strong scattering effect in the enamel caries lesion results in less transparency. The decreased light transmission associated with the lesion can be detected when compared to that of the surrounding sound tissue. 

The use of dental radiography should always be limited, even though it is the most often employed concept of routine examination. Dental radiographs also lack the sufficient ability for early caries detection [13, 16]. An initial caries lesion may be missed or underestimated in size in radiographs due to low attenuation of radiation in lesion, particular physical properties of the tooth structure, and imperfect technique such as overlapping. In contrast, the TI method offers the advantage of allowing for repeated projection to overcome some of these limitations. 

The importance of the location of the caries lesion and how the resolution differs when the resultant image has to traverse a thick part versus a thinner part of the tooth to reach the detector is also of interest. Contrast calculation of the signal generated by a single lesion located near versus far from the CCD camera can be estimated. The ratio between the contrasts of images captured from both sides of the tooth can estimate the more precise location of the caries lesion on the approximal surface.

## 4. Summary

Both QLF and the TI methods enable imaging detection of enamel caries that can be digitally stored and viewed later. The QLF method also includes image analysis software which measures the difference in fluorescence between sound and demineralised enamel. Changes in fluorescent radiance and lesion area can be followed over time, to measure lesion development. The method seems to have been rapidly adopted as a standard reference measure in clinical tests of the efficacy of preventive measures. 

Transillumination of enamel with NIR light is a promising technique for the detection and imaging of occlusal and approximal lesions. Application of repeatable, nonionising radiation of the tooth allows the TI method to be used without restriction to monitor the caries process. The method overcomes some of the limitations of dental radiography such as overlapping. Moreover, the method can indicate the relative position of a lesion on approximal surfaces by calculating the ratio of contrast values obtained by illuminating tooth from the lingual or buccal surface, respectively. The method uses a range of wavelengths where low-cost light sources are available and the transmitted image can be detected by an ordinary CCD camera, similar to the one in mobile phones. The method can therefore be developed at reasonable cost as a fibre optic probe for intraoral use, connected to an ordinary computer screen. 

The LF method generates a simple numerical index of de- and remineralisation in enamel and dentin that can be recorded in the patient's file and monitored over time. The instrument is easy to handle and can also be purchased at a reasonable price. New methods should be critically appraised according to strict criteria. Among LF studies there is a wide variation in specific design features (the number of teeth included, the threshold for LF scores, validation methods, the outcomes expressed, etc.). In vivo studies highlight the importance of rigorous clinical studies to confirm promising laboratory results. Results of the LF method in vivo have been somewhat contradictory. Therefore, The LF device should be regarded at most as a supplementary aid for detection of caries on coronal surfaces, pending the publication of further clinical studies.

## Figures and Tables

**Table 1:** Summary of optical caries detection methods.

Optical Coherence Tomography	OCT
Polarized Raman Spectroscopy	PRS
Polarization Sensitive Optical Coherence Tomography	PS-OCT
Fibre Optic Transillumination	FOTI and DiFOTI
Quantitative Light-induced Fluorescence	QLF
Laser-induced Fluorescence	LF
Transillumination with Near-Infrared light	TI-NIR
Infrared fluorescence	IR fluorescence
Near-Infrared reflectance imaging	NIR reflectance imaging
Terahertz Pulse Imaging Multiphoton imaging	TPI
Time-Correlated Single-Photon Counting Fluorescence Lifetime Imaging	TCSPC FLIM
